# Distinct Neurocognitive Strategies for Comprehensions of Human and Artificial Intelligence

**DOI:** 10.1371/journal.pone.0002797

**Published:** 2008-07-30

**Authors:** Jianqiao Ge, Shihui Han

**Affiliations:** 1 Department of Psychology, Peking University, Beijing, People's Republic of China; 2 Functional Imaging Center, Peking University, Beijing, People's Republic of China; 3 Key Laboratory of Machine Perception (Ministry of Education), Peking University, Beijing, People's Republic of China; Victoria University of Wellington, New Zealand

## Abstract

Although humans have inevitably interacted with both human and artificial intelligence in real life situations, it is unknown whether the human brain engages homologous neurocognitive strategies to cope with both forms of intelligence. To investigate this, we scanned subjects, using functional MRI, while they inferred the reasoning processes conducted by human agents or by computers. We found that the inference of reasoning processes conducted by human agents but not by computers induced increased activity in the precuneus but decreased activity in the ventral medial prefrontal cortex and enhanced functional connectivity between the two brain areas. The findings provide evidence for distinct neurocognitive strategies of taking others' perspective and inhibiting the process referenced to the self that are specific to the comprehension of human intelligence.

## Introduction

Since the computer system dubbed “Deep Blue” won the chess game against the reigning world champion Garry Kasparov in 1996, human intelligence (HI) has been strongly challenged by artificial intelligence (AI). The development and application of intelligent robots has compelled humans to deal with both HI and AI in real life situations [1] and thus provokes questions of whether the human brain comprehends HI and AI in essentially the same way. During the last few decades, debates among philosophers, psychologists, and computer scientists have focused on whether HI and AI are subserved by similar computational processes [2]–[4]. However, few researchers have inquired whether the human brain employs homologous neurocognitive strategies to comprehend HI and AI.

Primate brains primarily evolved in adaptation to social complexity so as to interpret mental states and predict behaviors of conspecifics [5]. Psychological and brain imaging research suggests that humans may understand mental processes of other individuals by simulation [6]–[9], which demands taking others' perspectives [10], [11] and inhibiting one's own perspective [12], [13]. However, knowing that the appropriateness and effectiveness of computers and robots depends upon the program embedded [14], humans, as designers and users of AI, may comprehend a robot by analyzing its actions rather than by simulating its ‘mind’ [15]. On these grounds, we hypothesize that taking others' perspective and meanwhile inhibiting the process referenced to the self characterize the unique neurocognitive mechanisms of understanding HI in comparison with AI.

**Figure 1:**
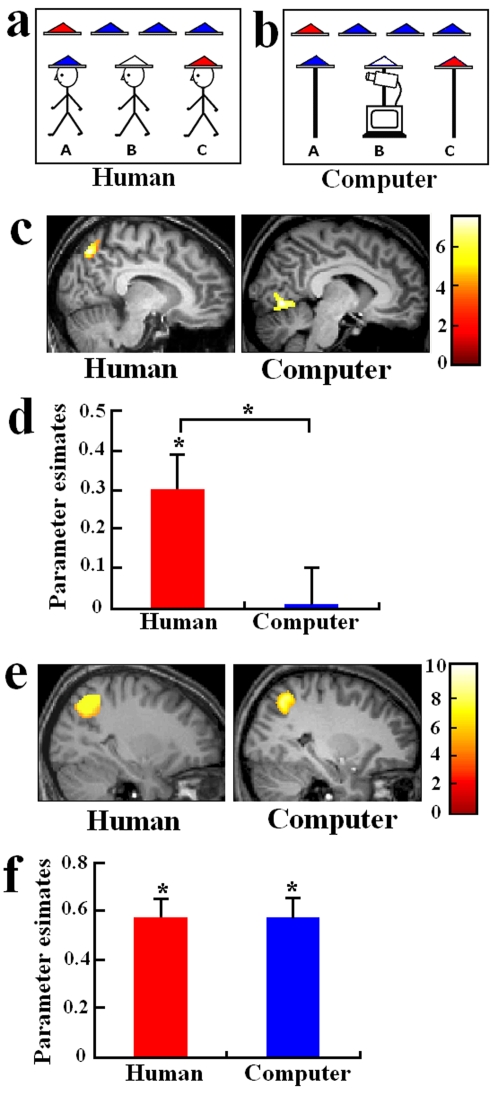
Illustration of stimuli and neural activity specific for PC and DR tasks. (a) and (b) Illustration of stimulus displays used in the MI and DR tasks. In this case, the human agent B and the computer received information about the color of the hat on Agent A or Rod A. Subjects were asked to judge if Agent B or the computer could infer or compute the color of his own hat given the existence of one red hat and three blue hats. (c) Activation shown in the contrast of PT vs. PC tasks. The contrast of PT vs. PC tasks in association with the human agent showed greater activation in the precuneus whereas the same contrast in association with the computer showed increased activation in the visual cortex. (d) Parameter estimates of signal intensity obtained in the precuneus ROI. The precuneus signal intensity was significantly greater with the human agent than with the computer. (e) Illustration of the activation in the right intraparietal sulci shown in the contrast of DR vs. PC tasks in association with the human agent and the computer. (f) Parameter estimates of signal intensity obtained in the right intraparietal ROI. The intraparietal signal intensity did not differ between the human agent and the computer.

Because reasoning is one of the core processes of intelligence [16], [17], we test our hypothesis by examining neural substrates involved in the comprehension of reasoning processes conducted by human agents and by computers. We developed a paradigm to assess whether the neural correlates of inference of other intelligence differ as a function of the agents affording the intelligence (human or computer). In this paradigm, subjects were informed of the following context before the study: 4 or 5 red or blue hats are available, and three of these hats are randomly assigned to three agents, as illustrated in Figure 1a. Agent B is provided with the contextual information (i.e., the number and color of hats available) and is able to see only the hat worn by the agent in front of him (i.e., Agent A in Figure 1a). Agent B guesses the color of his own hat given the information available to him. Subjects were asked to infer Agent B's reasoning and to judge whether Agent B is able to know the color of his own hat (a mental inference (MI) task). To contrast the MI task related to HI versus AI, another set of stimuli was designed in which the human agent is replaced by a computer that is connected to a camera, which shoots a picture of the hat in front of it (Figure 1b). Subjects were informed that the computer uses a program to compute the color of the hat and had to judge if the computer, which represents AI with the ability to conduct calculation and reasoning, is able to report the color of the hat on top itself given the information available. The information provided to the human agent and to the computer was identical. Moreover, the reasoning processes conducted by the human agent and by the computer can be formalized using the same algorithm, such that the only difference between HI and AI is the agent affording the reasoning processes. A between-subject design was used to avoid the interference between different strategies applied for HI and AI. 28 subjects were scanned, using functional magnetic resonating imaging (fMRI), while they performed the MI task. Half of the subjects were randomly assigned with human agent and the other half was assigned to the computerized agent, so as to reveal the neural substrates differentiating the inference of reasoning processes conducted by human agents and computers.

Previous research showed that taking a third-person perspective engages the precuneus [10], [11] whereas taking first-person perspective [11] and conducting self-referential processing [9], [18]–[20] recruits the ventral medial prefrontal cortex (vMPFC). Thus we were particularly interested in variations of activity in the precuneus and vMPFC in association with the inference of the reasoning processes in HI and AI. This was examined by contrasting the MI task with a deductive reasoning (DR) task based on the first-person perspective, in which subjects made inference of the color of Agent B's hat in Figure 1a or the color of the computer's hat in Figure 1b. A perspective-taking (PT) task of judging the color of the hat that Agent B can see or the camera on the computer can shoot was used to identify the neural correlates involved in perspective taking. A perception (PC) task of judging the color of Agent B's hat was used to control for low-level perceptual processing (see **Figure S1**).

## Materials and Methods

### fMRI study

#### Participants

Twenty-eight native Chinese undergraduate and graduate students participated in this study as paid volunteers. Fourteen (6 males, 8 females; aged between 18 and 28, mean age 23.36±2.87 years) were assigned with human intelligence (HI) and fourteen (6 males, 8 females; mean age 24.12±2.35 years, aged between 19 and 26) with artificial intelligence (AI). Participants' gender and education were matched in the two subject groups. All participants were right-handed and had no neurological or psychiatric history. All had normal or corrected-to-normal vision and were not color blind. Written informed consent was obtained from each participant prior to scanning. This study was approved by the Local Ethic Committee of the Department of Psychology, Peking University.

#### Stimuli and Procedure

The stimulus displays were presented through a LCD projector onto a rear-projection screen located at a subject's head, viewed with an angled mirror positioning on the head-coil. Visual stimuli consisted of colorful pictures, as illustrated in Figure S1. Each stimulus display subtended a visual angles of 16.2×13.5° (width and height) at a viewing distance of 90 cm. Each stimulus display of human intelligence (HI) consisted of three human agents (A, B, and C) who stand in a queue and all face to the left or right. Participants were informed of the following contextual information before the scanning procedure. There are 4 or 5 red or blue hats, as those shown above the agents in Figure S1. Agent B knows the number and color of hats available and that three of the hats are randomly assigned to the agents. Agent B can only see the hat worn by the agent in front of him.

Four tasks were designed based on the stimuli (Figure S1). (1) Perception task (PC), to identify the color of B's hat; (2) Perspective taking task (PT), to identify the color of the hat that B sees; (3) Deductive reasoning task (DR), to make inference of the color of B's hat; (4) Mental inference task (MI), to identify whether B is able to know the color of his own hat. The color of B's hat was not shown in the stimulus displays used in DR and MI tasks to make sure that participants must use the context information to make inference of the actual color of B's hat or the reasoning processes conducted by Agent B. To match the visual features of the stimuli used in PC/PT and DR/MI tasks, the color of C's hat was not given in the stimulus displays used in PC and PT tasks.

All aspects of the stimuli and tasks with AI were identical to those with HI except the following. The hats were hung on three rods rather than human agents. In addition, participants were informed that there is a camera installed on Rod B, which is connected to a computer below. The camera can only a picture of the hat in front of it. The computer can run a program to compute the color of the hat hung on Rod B when the information it receives from the camera and the context information are sufficient. In the PT task, participants were instructed to identify what color of the hat the camera shoots. In the MI task, participants were asked to identify whether the computer could compute the color of the hat hung on Rod B based on the information it receives through the camera and the context information provided in a stimulus display. The instructions in the PC and DR tasks were identical with HI and AI stimuli.

A box-car design was used. Each task session repeated ten times and were evenly distributed in five scans. Each task session was preceded by an instruction of 4 s that identified the task, which was followed by 4 trials. On each trial, a stimulus display was presented for 3500 ms under the question and was followed by a 500 ms interval. Two task sessions were separated by a fixation cross (0.36×0.36°) of 6 s at the center of the screen. Participants pressed one of the two buttons with the right index or middle finger to answer the questions. The order of the tasks and the assignment of response buttons were counterbalanced across scans and participants.

#### Behavioural Data Analysis

Response accuracy and reaction time (RT) were recorded to each task and subjected to a one-way analysis of variance (ANOVA) with Task (MI, DR, PT, PC) as the main effect (Figure S2). Separate t-tests were also conducted to compare the difference in behavioural performances between each two tasks.

#### fMRI Data Acquisition

Scanning was performed on a 3T Siemens Trio system using a standard head coil at Beijing MRI Center for Brain Research. 32 transversal slices of functional images that covered the whole brain were acquired using a gradient-echo echo-planar pulse sequence (64×64×32 matrix with 3.4×3.4×4.4-mm spatial resolution, TR = 2000 ms, TE = 30 ms, FOV = 220 mm, flip angle = 90°). After the functional scanning, anatomical images were obtained using a standard 3D T1-weighted sequence (256×256×176 matrix with 0.938×0.938×1.3-mm spatial resolution, TR = 1600 ms, TE = 3.93 ms).

#### fMRI Data Analysis

SPM2 (the Wellcome Department of Cognitive Neurology, UK) was used for data processing and analysis. The blood oxygen level dependence (BOLD) functional images were realigned to the first scan to correct for the head movement between scans. The anatomical image was co-registered with the mean functional image produced during the process of realignment. All images were normalized to a 2×2×2 mm^3^ Montreal Neurological Institute (MNI) template in Talairach space using bilinear interpolation. Functional images were spatially smoothed using a Gaussian filter with a full-width at half maximum (FWHM) parameter for 8 mm. The image data were modeled using a box-car function. Parameter estimates for each condition were calculated from General Linear Model (GLM) based on hemodynamic response funciton Contrasts were calculated between each two conditions. Statistical effects were first assessed in individual subjects using a fixed effect analysis. Random effect analyses were then conducted based on statistical parameter maps from each individual subject to allow population inference. A one-sample t-test was applied to determine group activation for each effect. Significant activation was identified at the cluster level for values exceeding a P value of 0.05 (corrected for multiple comparisons). The SPM coordinates for a standard brain from MNI template were converted to Talairach coordinates using a nonlinear transform method (http://www.mrc-cbu.cam.ac.uk /Imaging/mnispace.html). To compare the neural activities with HI and AI, parameter estimates of signal intensity were extracted from region of interests (ROIs) and compared using two-sample t-tests. The ROI in the precuneus was defined as a sphere with 6 mm diameter centered at the peak voxel observed in the contrast of PT vs. PC tasks and the ROI in vMPFC was defined as a sphere with 6 mm diameter centered at the peak voxel of vMPFC activation (BA10, 0/49/7) associated with self-referential processing observed in our previous research [19], [20]. The ROI in the intraparietal sulcus was defined as a sphere with 6 mm diameter centered at the peak voxel observed in the contrast of DR vs. PC tasks.

#### Psychophysiological Interaction Analysis

After we identified the involvement of several brain areas in the MI task, we conducted a psychophysiological interaction (PPI) analysis [21] to examine the covariation between the neural activities in two brain areas. We were particularly interested in the brain areas that showed variation of functional connectivity with the ventral medial prefrontal cortex during the MI task. The coordinates of the peak voxel in the cluster identified in the random effect analysis comparing the MI task with the DR task were used to serve a landmark for the individual seed voxels. An ROI of a sphere with 10 mm diameter in the dorsal as well as in the ventral medial prefrontal cortex was searched around the peak voxel. The time series of the signals of each ROI were then extracted and PPI regressor was calculated as the element-by-element product of the mean-corrected activity of this ROI and a vector coding for these two differential task effects. The PPI regressor reflected the interaction between psychological variable (e.g., MI vs. DR) and the activation time course of the seed region (i.e., the ventral medial prefrontal cortex). The individual contrast images reflecting the effects of this interaction from 14 participants were subsequently subject to a one-sample t-test. The brain regions that showed increased functional connectivity with the seed ROI were identified with threshold of p<0.05 (corrected) at the cluster level in the group analysis. Parameter estimates of signal intensity were extracted from the precuneus ROI with HI and AI stimuli and subjected to two-sample t-tests.

### Behavioral study

#### Participants

Thirty-two native Chinese undergraduate and graduate students participated in this study as paid volunteers (16 males, 16 females, aged between 19–26). 16 subjects were assigned with HI stimuli (8 males, 8 females; mean age 20.81±1.80 years) and 16 subjects with AI stimuli (8 males, 8 females; mean age 22.06±2.08 years). Participants' gender and education were matched in the two subject groups. All participants were right-handed and had no neurological or psychiatric history. All had normal or corrected-to-normal vision and were not color blind. None of the participants had attended the current MRI study. This study was approved by a local ethic committee at the Department of Psychology, Peking University.

#### Stimuli and Procedure

Visual stimulus of HI and AI were identical to those used in the MRI experiment. The stimulus were presented in the center of an 18-inch color monitor, subtended a visual angle of 16.2×13.5° (width and height) at a viewing distance of 60 cm. Subjects performed only the mental inference task, i.e., to infer whether Agent B or the computer is able to report the color of his own hat given the information available.

Each subjects participated in two blocks of 48 trials. On half of the trials Agent B or the computer could infer the color of his own hat (‘Yes’ response) whereas, on the other trials, Agent B or the computer could not infer the color of his own hat (‘No’ response). On half of the ‘No’ response trials, neither subjects nor Agent B could infer the color of B's hat (consistent condition). On the other ‘No’ response trials, subjects could infer the color of B's hat but Agent B or the computer could not (inconsistent condition). On each trial, a stimulus display was presented until the subjects made a response, which was followed by fixation cross with a duration varying randomly between 750 ms and 1750 ms. Subjects responded to each stimulus by a button press using the left and right index finger. The assignment of the left or right index finger to ‘Yes’ and ‘No’ responses was counterbalanced across subjects.

## Results

The mean response accuracy across all tasks was 95.2% and 91.3% related to HI and AI stimuli, respectively. A one-way analysis of variance (ANOVA) of reaction times (RTs) showed a significant main effect of Task (HI: F(3,39) = 151.8, p<0.0001; AI: F(3,39) = 152.5, p<0.0001, **Figure S2**). Paired t-tests confirmed that RTs to the PC task were shorter than those to the PT, DR, and MI tasks (all p<0.002). Furthermore, RTs to the PT task were shorter than those to the DR and MI tasks (all p<0.0001). However, there was no significant difference in RTs between the DR and MI tasks (p>0.05).

fMRI data analysis first identified neural correlates of perspective taking by contrasting PT vs. PC tasks in association with HI, which uncovered increased blood-oxygen-level dependent (BOLD) activity in the precuneus (Brodmman area (BA) 7, Talairach coordinates: 8/−65/51, Z = 4.60, voxel number = 354, and −10/−60/50, Z = 4.10, voxel number = 129, both p<0.05, corrected for multiple comparisons, Figure 1c, d). However, the precuneus activation was not observed with the PT task when assessing AI. The difference in precuneus activation linked to PT task between HI and AI was verified by two-sample t-tests comparing contrast values of signal intensity in the region of interest (ROI), a sphere with a 6 mm diameter centered at the peak voxel of the precuneus activation (t = 2.285, p = 0.03). However, the PT task with AI resulted in increased activity in the visual cortex (BA18, –6/−70/2, Z = 4.34, voxel number = 432, p<0.001, corrected), suggesting enhanced visual analysis of the stimuli when dealing with AI. The contrast of DR vs. PC tasks applied to both HI and AI revealed neural correlates of deductive reasoning in bilateral intraparietal sulci (BA 7, HI: −28/−52/39, Z = 5.40, voxel number = 1020, and 33/−62/40, Z = 4.71, voxel number = 1574; AI: −34/−50/43, Z = 5.28, voxel number = 899, and 28/−56/44, Z = 4.88, voxel number = 785; all p<0.001, corrected, Figure 1e, f), and the magnitudes of DR-related parietal activities did not differ between HI and AI (t = 0.007; p = 0.994). These results are consistent with previous observations that the precuneus is involved in taking third-person perspective in space [11] and that the posterior parietal cortex subserves mental calculation and reasoning [22]–[25].

**Figure 2:**
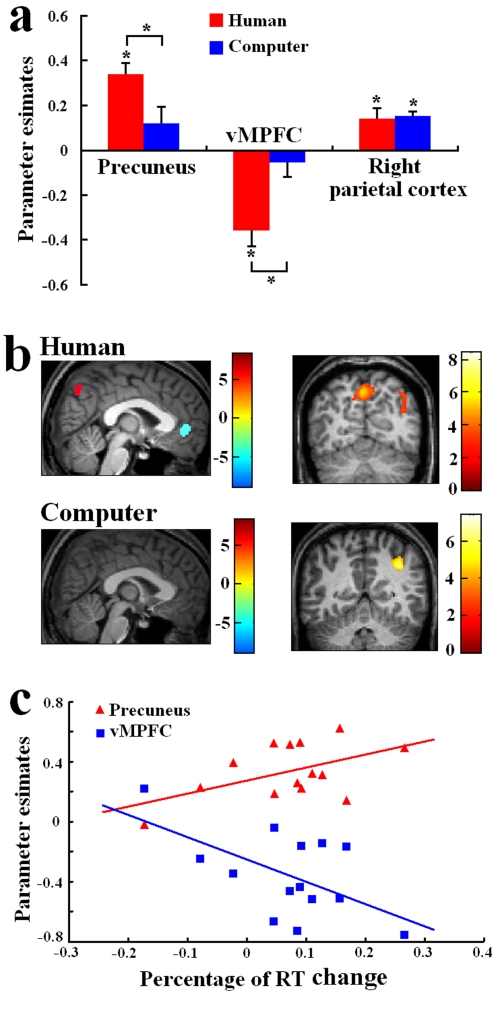
Neural activity specific for the MI task. (a) Parameter estimates of signal intensity for suprathreshold voxels from the contrast of MI vs. DR tasks with human agents and computers; (b) T-Map projected on a single subject anatomical structure for the contrast of MI vs. DR tasks with HI. Activations in the precuneus and intraparietal sulcus and deactivation in vMPFC were observed. Activation was observed only in the intraparietal sulcus in the contrast of MI vs. DR tasks with AI. Color bars show scales of t-values; (c) Correlation between RT changes and the precuneus/vMPFC activity associated with the MI task with HI.

To assess whether the inference of reasoning processes of human agents is characterized with enhanced processing of perspective taking and inhibition of self-referential processing, we compared signal intensity in the precuneus and vMPFC associated with the MI and DR tasks. The ROI in the precuneus was centered at the peak voxel observed in the contrast of PT vs. PC tasks (BA7, 8/−65/51) and the ROI in vMPFC was centered at the peak voxel of the vMPFC activation (BA10, 0/49/7) associated with self-referential processing observed in the previous research [19], [20]. Relative to the DR task, the inference of reasoning processes of human agents gave rise to increased BOLD signal intensity in the precuneus but decreased BOLD signal intensity in vMPFC (precuneus: t = 6.957, p = 0.000; mPFC: t = −3.654, p = 0.002, Figure 2a, b). Nevertheless, the inference of reasoning processes of computers failed to modulate precuneus (t = 1.606, p>0.1) or vMPFC (t = −0.719, p>0.4) activity relative to the DR task. Two-sample t-tests confirmed the different patterns of precuneus and vMPFC activations linked to the MI task between HI and AI (precuneus: t = 2.486, p = 0.02; vMPFC: t = −2.897, p = 0.008). Relative to the DR task, the MI task also induced increased activity in the right intraparietal sulcus when perceiving both HI (t = 2.881, p = 0.013) and AI (t = 7.083, p = 0.000, Figure 2a, b). However, the parietal activity associated with the MI task did not differ between HI and AI (two-sample t-tests: t = −0.213; p = 0.834), suggesting that inference of reasoning processes of human agents and computers engaged similarly enhanced processes of mental calculation and reasoning.

To examine how the cognitive strategies specific to the inference of reasoning processes of human agents influences individual behavioral performances, we calculated the correlation between signal intensities in the precuneus and the vMPFC and the percentage of RT change in the MI relative to DR tasks. We found that RT variations correlated positively with precuneus signal intensity (r = 0.528, p = 0.05) but correlated negatively with signal intensity in the vMPFC (r = −0.576, p = 0.03, Figure 2c), suggesting that recruitment of others' perspective and self-inhibition results in delayed behavioral performance in the MI task.

**Figure 3:**
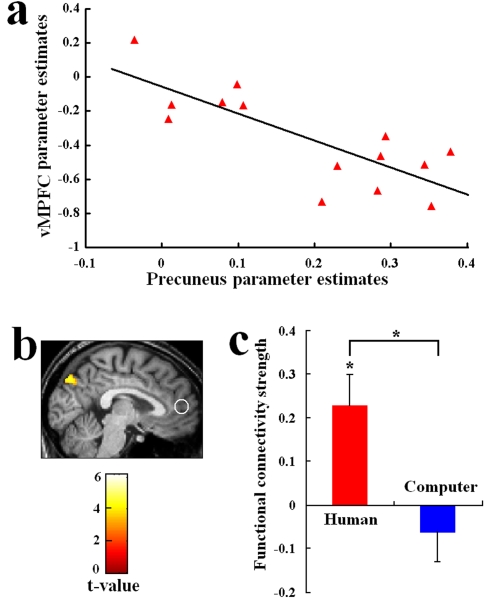
Functional connectivity associated with the MI task. (a) Correlation between the neural activities in the precuneus and vMPFC during the MI task with HI; (b) The PPI analysis showed increased functional connectivity between the precuneus and vMPFC in the MI compared with DR tasks with HI. An ROI was defined in vMPFC and brain areas showed correlation the vMPFC activity was searched in the whole brain. The color bar shows scales of t-values; (c) Parameter estimates of the functional connectivity strength. The functional connectivity strength between the precuneus and vMPFC was stronger with HI than AI.

As taking others' perspective requires resisting interference from processing of stimuli from the self-perspective [12], these two processes should coordinate inversely with each other during the inference of reasoning processes of human agents. Indeed, we found a significant negative correlation between the neural activities in the precuneus and the vMPFC linked to the MI task with HI (r = −0.794, p = 0.001, Figure 3a), indicating that subjects who recruited more perspective-taking were also more likely to inhibit self-referential processing. Because the correlation implies the existence of functional connectivity between the two areas, we conducted a psychophysiological interaction (PPI) analysis [25] to assess covariations between the neural activity in the two brain areas. The PPI analysis confirmed enhanced functional connectivity between the precuneus and vMPFC during the MI relative to the DR task (Figure 3b). However, such enhanced functional connectivity was evident with HI (p<0.05, corrected) but not with AI. The difference in functional connectivity strength between the precuneus and vMPFC was confirmed between HI and AI using two-sample t-test (t = 3.322, p = 0.006, Figure 3c).

**Figure 4:**
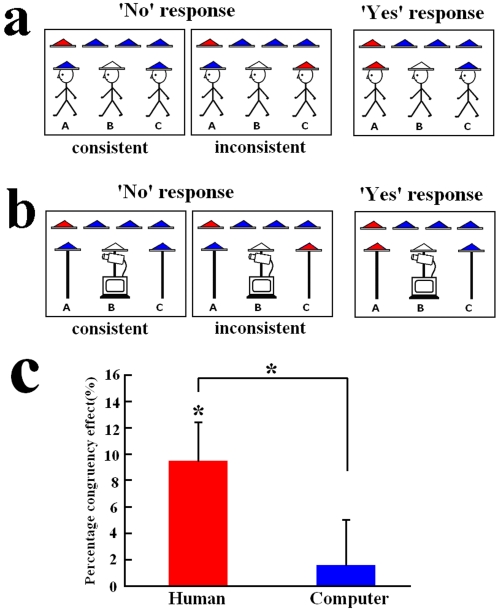
Illustration of stimuli and behavioral results in the independent behavioural study. (a) and (b) Illustration of stimulus displays used in the independent behavioural study. Subjects were asked to infer whether Agent B or the computer knows the color of his own hat; (c) The percentage congruency effect. This was defined as the percentage variation of reaction times in the inconsistent condition relative to those in the consistent condition ((RT_inconsistent_−RT_consistent_)/RT_consistent_), which was greater with HI than with AI.

The hypothesis that the inference of reasoning processes of human agents engages taking another's perspective while inhibiting self-referential processing predicts that, relative to situations when the information obtained from the first- and third-person perspective is consistent, inconsistencies between the information obtained from the first- and third-person perspective may recruit additional neural process to overcome the conflict during the MI task and thus slow down behavioural responses. To verify this, we conducted an independent experiment to measure RTs in the MI task with both HI and AI (see supplementary methods). We were particularly interested in RTs of ‘No’ responses that could be classified into two categories: (1) *Consistent*: the information obtained from the first- and third-person perspective was consistent and neither subjects nor Agent B could conclude the color of B's hat based on the contextual information; (2) *Inconsistent*: the information obtained from the first- and third-person perspective was inconsistent and subjects could conclude the color of B's hat but Agent B could not. If subjects took Agent B's perspective and inhibited their self-perspective during mental inference, responses should be slower in the inconsistent rather than consistent conditions. RTs in each condition for HI and AI groups were shown in Table S1. To normalize the individual differences of response speeds, the percentage congruency effect ((RT_inconsistent_−RT_consistent_)/RT_consistent_) was calculated to index the RT variation between *Consistent* and *Inconsistent* conditions. There was a significant congruency effect with HI (9.36%±2.81%, t = 3.33, p = 0.005), suggesting that incongruent information from the first- and third-person perspective slowed ‘No’ responses. However, no reliable congruency effect was observed with AI (1.58%±3.51%, t = 0.216, p = 0.832), indicating that incongruent information from the first- and third-person perspective did not affect ‘No’ response speed when subjects inferred reasoning processes conducted by computers (Figure 4). Two-sample t-test confirmed the difference in congruency effect between HI and AI groups (t = 2.070, p = 0.047).

## Discussion

From a programmer's perspective, the same algorithm can be used to describe the reasoning processes conducted by of both human agents and computers. However, our fMRI results demonstrate that the inference of reasoning processes engages distinct neurocognitive strategies in the human brain depending upon the agents affording the reasoning processes. Relative to deductive reasoning based on the first-person perspective, the inference of reasoning processes conducted by human agents was associated with increased activity in the precuneus but decreased activity in vMPFC. Interestingly, this pattern was not observed during the inference of the reasoning processes conducted by computers. Given the functional role of the precuneus in taking others' perspective [10], [11] and of the vMPFC in self-referential processing [9], [18]–[20], our findings indicate that the inference of HI is characterized by enhanced processes of taking others' perspective and inhibiting one's own perspective and self-referential processing compared with the inference of AI. Additionally, the results of the correlation analysis demonstrated that the amount of precuneus and vMPFC activity contributed to the response speeds of the inference of reasoning processes of human agents, suggesting a pivotal role of perspective taking and self inhibition in modulation of behavioral performance when interpreting HI.

The contrast of PT vs. PC tasks in our study also provided evidence for the involvement of the precuneus in perspective taking, consistent with previous observations [10], [11]. However, this was evident when subjects dealt with HI but not with AI. Judging what information a computer could process through a camera may be conducted by inspecting visual details of the stimuli, consistent with the enhanced visual activity in the PT task during assessment of AI. The precuneus and vMPFC activity correlated negatively and demonstrated significant coordination as revealed by enhanced functional connectivity between the two substructures. This supports the idea that taking another's perspective and inhibiting self-perspective related to the MI task are two processes that function in coordination [26]. Taking others' perspective may weaken self-referential processes and induce decreased vMPFC activity. Interestingly, the negative correlation and enhanced functional connectivity between the precuneus and the vMPFC were observed during the inference of the reasoning process associated with human agents but not with computers, providing further evidence for the unique neurocognitive strategies involved during coping with HI.

The dissimilar patterns of precuneus and vMPFC activity associated with HI and AI could not arise from general changes of brain activity related to arousal because other brain areas such as the right posterior parietal cortex, which reflects the recruitment of additional mental calculation and reasoning processes in the MI task, showed comparable magnitudes with HI and AI. Nor could the differential neural activity be elucidated by task difficulty, which was well controlled as RTs to the MI and DR tasks were comparable. The changes in the precuneus and vMPFC activity associated with the inference of reasoning processes of human agents showed a reverse pattern of variation and thus could not have been induced simply by changes of baseline activity in the default mode network since both medial frontal and parietal regions tend to decrease their activity during cognitively demanding tasks [27]. The precuneus activity cannot be attributed to enhanced deductive reasoning [28] in the MI compared with the DR task because no increased precuneus activity was observed with AI although the task demands (e.g., complexity of reasoning) and magnitudes of cognitive load were comparable in both HI and AI tasks.

Our findings shed light on the social nature of neurocognitive mechanisms underlying comprehension of HI. Taking others' perspective and inhibiting self-referential processing may evolve to improve efficiency and accuracy of inference of mental states of conspecifics. In agreement with this, subjects showed higher response accuracy in the MI task with HI than with AI (95.0% vs. 86.3%; t = 3.580, p = 0.002) but performed equally well with HI and AI in the DR Task (93.1% vs. 89.0%, t = 1.361, p = 0.185). Subjects did not adopt these strategies when dealing with AI possibly because humans treat a computer as an extended part of the self and thus do not differentiate the information perceived by the self and the information owned by the computer. This may then result in more errors during the inference of reasoning processes of computers. Lack of these cognitive strategies can lead to deficits in social interactions, such as that seen in autism [26], where individuals demonstrate a difficulty to cope with other humans [29] and fail to show suppressed vMPFC activity during cognitively demanding tasks [30]. However, people with autism enjoy interacting with computers [31] and playing with robots [1]. This may be interpreted by assuming that autistic patients do not have to take a robot's perspective and inhibit self-referential processing during their interactions with robots or computers.

Previous studies of mental attribution have commonly identified neural correlates of mental attribution by comparing two episodes describing consecutive events, using language or cartoons [32], [33]. In these studies, subjects performed a mental attribution task with one episode but a non-mental task with the other. The contrast of the two tasks revealed increased activity in a neural network including the dorsal MPFC [34]–[36] and temporoparietal junction [37]. The paradigm used in the current work excluded any differences between the MI and DR tasks in the processing of language, biological motion, and causal coherence of visual events that are not domain specific for inference of human mental states. The comparison between neurocognitive mechanisms engaged in the inference of the reasoning processes of human agents and computers helped to highlight domain-specific neurocognitive processes related to inference of human mental processes. The contrast of MI vs. PC tasks showed increased activation in the dorsal MPFC when subjects dealt with both HI and AI (**Figure S3**), consistent with the previous work [34]–[36]. However, the dorsal MPFC activity did not differ between MI and DR tasks at the threshold of p<0.05 (corrected for multiple comparisons), suggesting that the dorsal MPFC activity was not specific to the inference of human mental processes. By contrast, we showed here that the precuneus activation, the vMPFC deactivation, and the enhanced functional connectivity between the two brain areas characterize the unique neurocognitive processes involved in the inference of HI.

Our findings complement recent research on the effect of biological agency on neural responses involved in social cognition. For instance, relative to the prediction of a computer's action, forecasting a person's finger movements activated a neural circuit consisting of the MPFC, superior temporal sulcus, and Broca's area [38]. In an ultimatum game in which one player decided how to split a sum of money with another player, human subjects rejected unfair offers from human partners at a higher rate than those made by a computer. The behavioral difference was associated with increased activation in bilateral anterior insula, dorsolateral prefrontal cortex, and anterior cingulate cortex [39]. It is not surprising that human brains utilize specific neurocognitive mechanisms to assign finger movements and to produce emotional responses to a person relative to a computer given that prediction of movements of body parts and moral judgment can only be applied to human agents. The current study extends the previous research by showing that, although HI and AI may use the same algorithms to conduct reasoning processes, human brains employ distinct neurocognitive strategies to deal with the two forms of intelligence possibly because, in the human mind, the relationship between humans (i.e., conspecifics) and the relationsip between humans and AI (i.e., creator vs. creature) are essentially different.

In summary, we have shown that that the inference of reasoning processes of human agents is underpinned by a unique pattern of neural activition including increased precuneus activity, decreased vMPFC activity, and enhanced functional connectivity between the two brain areas. These fMRI results lend support to the hypothesis that comprehension of HI engages two key process, i.e., taking others' perspective and inhibiting self-referential process. These neurocognitive processes are not involved in the inference of reasoning processes conducted by a computer, highlighting the essential difference in neurocognitive strategies used to cope with HI versus AI, which shed new light on future research of human-robot interactions.

## Supporting Information

Figure S1 Illustration of stimuli and procedure of the fMRI study. (a) and (b) Illustration of the stimulus displays showing human agents or computers. Instructions for each task are shown below each stimulus display. (c) Illustration of the block design of the current study. Each block of 20s consisted of 4 trials preceded by a 4s instruction. Two neighboring blocks were separated by a 6s interval during which only a fixation cross was displayed.
(0.15 MB TIF)

Figure S2 Behavioral performance in the fMRI study. Reaction times to the MI, DR, PT, and PC tasks with the human agent. (b) Reaction times to the MI, DR, PT, and PC tasks with the computer.
(0.06 MB TIF)

Figure S3 Dorsal MPFC activation in association with the MI task linked to human and artificial intelligence. (a) The dorsal MPFC activation shown in the contrast of MI vs. PC tasks in association with the human agent (BA8/32, −6/14/50, Z = 3.96, voxel number = 352). (b) The dorsal MPFC activation shown in the contrast of MI vs. PC tasks in association with the computer (BA8, −4/36/45, Z = 4.60, voxel number = 335).
(0.09 MB TIF)

Table S1 Mean RTs and response accuracy (±SD) of the behavioral study
(0.02 MB DOC)
