# Supplementary figures and images for: Distinct Neurocognitive Strategies for Comprehensions of Human and Artificial Intelligence
Source: PLoS One. 2008 Jul 30;3(7):e2797. doi: 10.1371/journal.pone.0002797 (PMC2453324; doi:10.1371/journal.pone.0002797)

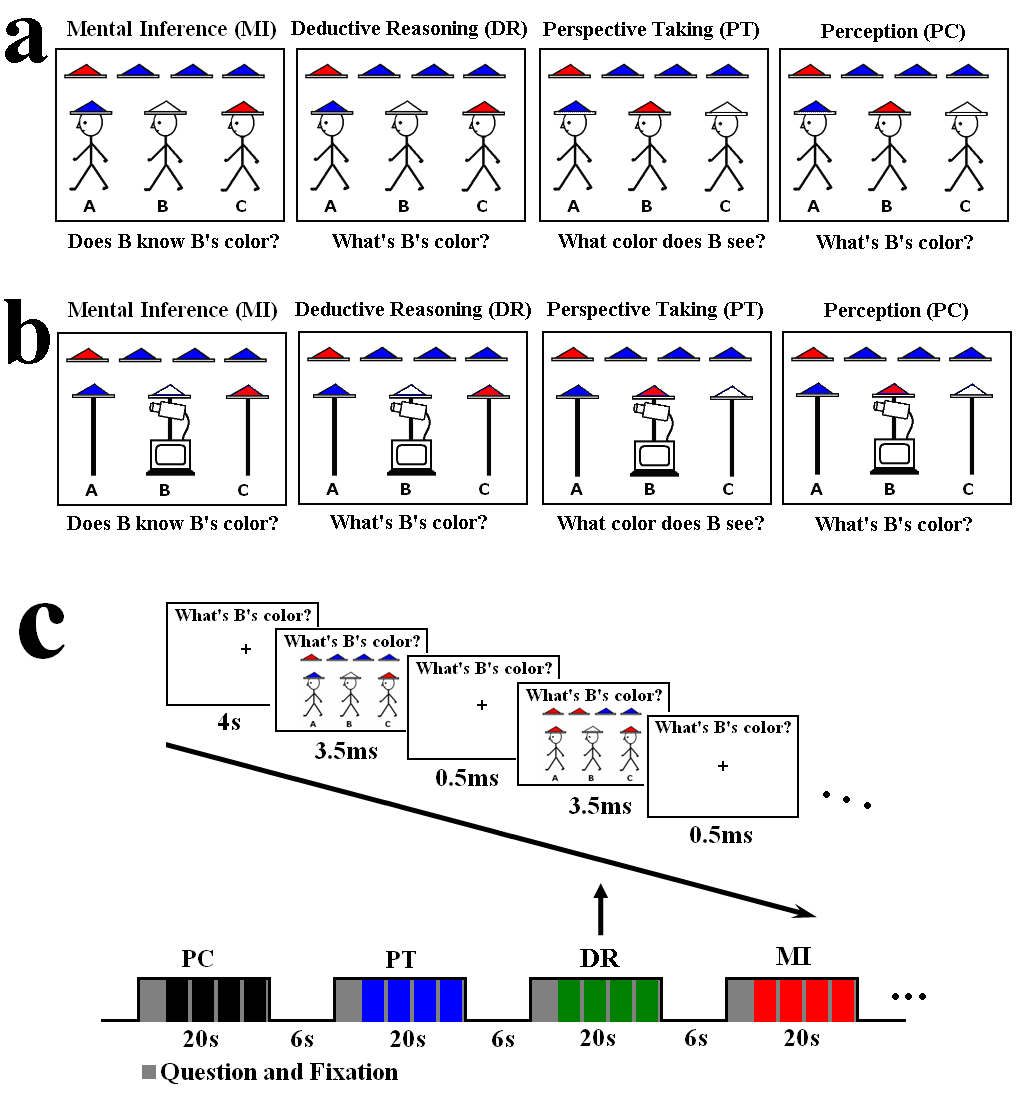

Supplement: Figure S1 — Illustration of stimuli and procedure of the fMRI study. (a) and (b) Illustration of the stimulus displays showing human agents or computers. Instructions for each task are shown below each stimulus display. (c) Illustration of the block design of the current study. Each block of 20s consisted of 4 trials preceded by a 4s instruction. Two neighboring blocks were separated by a 6s interval during which only a fixation cross was displayed. (0.15 MB TIF) [file pone.0002797.s001.tif]

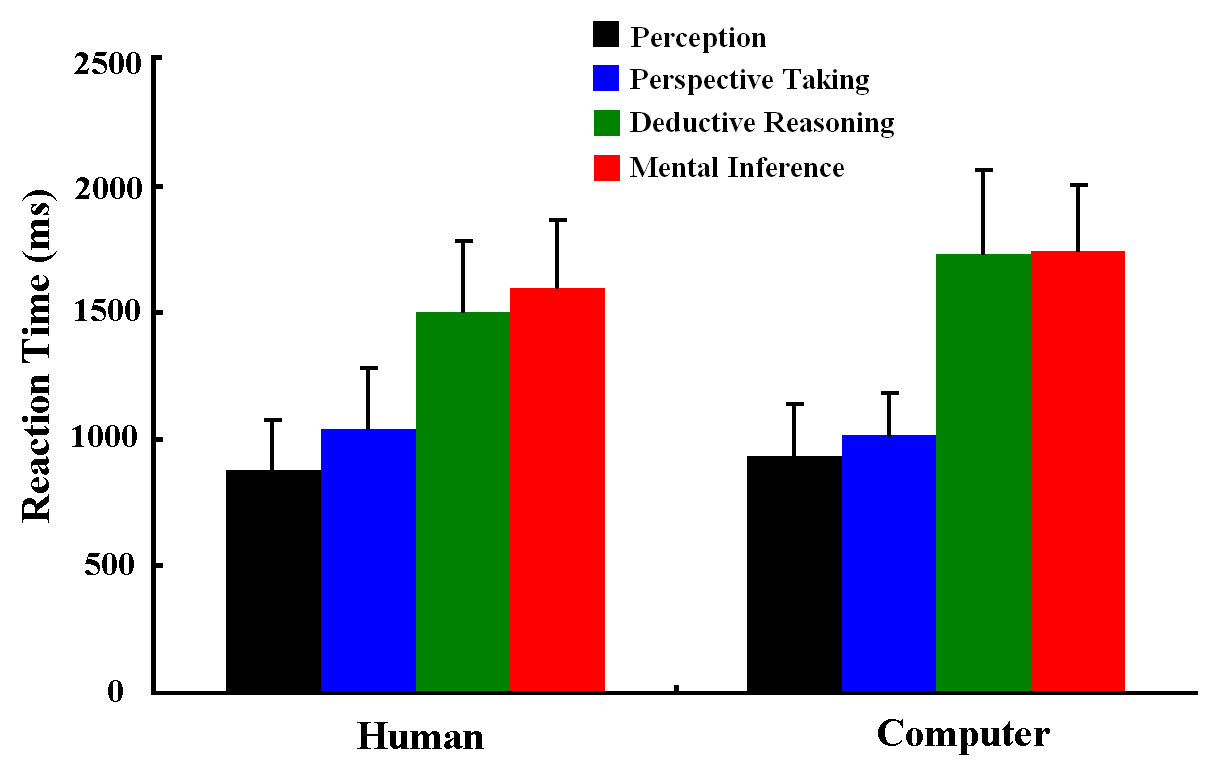

Supplement: Figure S2 — Behavioral performance in the fMRI study. Reaction times to the MI, DR, PT, and PC tasks with the human agent. (b) Reaction times to the MI, DR, PT, and PC tasks with the computer. (0.06 MB TIF) [file pone.0002797.s002.tif]

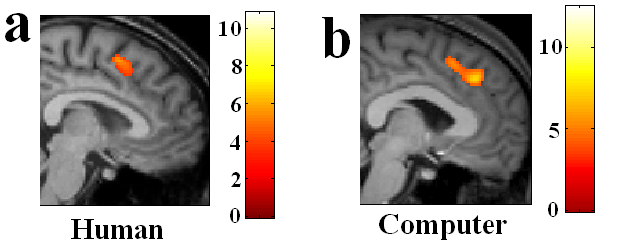

Supplement: Figure S3 — Dorsal MPFC activation in association with the MI task linked to human and artificial intelligence. (a) The dorsal MPFC activation shown in the contrast of MI vs. PC tasks in association with the human agent (BA8/32, −6/14/50, Z = 3.96, voxel number = 352). (b) The dorsal MPFC activation shown in the contrast of MI vs. PC tasks in association with the computer (BA8, −4/36/45, Z = 4.60, voxel number = 335). (0.09 MB TIF) [file pone.0002797.s003.tif]
