# Supplementary material for: Distinct Neurocognitive Strategies for Comprehensions of Human and Artificial Intelligence
Source: PLoS One. 2008 Jul 30;3(7):e2797. doi: 10.1371/journal.pone.0002797 (PMC2453324; doi:10.1371/journal.pone.0002797)
Supplement: Table S1 — Mean RTs and response accuracy (±SD) of the behavioral study (0.02 MB DOC) [file pone.0002797.s004.doc]

**Table S1**. Mean RTs and response accuracy (±SD) of the behavioral study

____________________________________________________________________

No-response (consistent) No-response (conflict) Yes-response

____________________________________________________________________

RTs (ms)

HI 1279 ± 237 1374 ± 259 1356 ± 300

AI 1874 ± 236 1866 ± 224 1559 ± 120

Accuracy (%)

HI 96.1 ± 1.1 94.8 ± 0.8 95.6 ± 0.8

AI 97.9 ± 1.1 96.4 ± 1.3 98.0 ± 0.6

____________________________________________________________________
